# Effect of Vitamin D Treatment on Dynamics of Stones Formation in the Urinary Tract and Bone Density in Children with Idiopathic Hypercalciuria

**DOI:** 10.3390/nu12092521

**Published:** 2020-08-20

**Authors:** Joanna Milart, Aneta Lewicka, Katarzyna Jobs, Agata Wawrzyniak, Małgorzata Majder-Łopatka, Bolesław Kalicki

**Affiliations:** 1 Clinic of Paediatrics, Nephrology and Paediatric Allergology, Military Institute of Medicine, 128, 04-141 Warsaw Szaserów, Poland; jfurgal@wim.mil.pl (J.M.); kjobs@wim.mil.pl (K.J.); awawrzyniak@wim.mil.pl (A.W.); kalicki@wim.mil.pl (B.K.); 2 Laboratory of Food and Nutrition Hygiene, Military Institute of Hygiene and Epidemiology, Kozielska, 4, 01-163 Warsaw, Poland; 3 The Main School of Fire Service, Slowackiego 52/54, 01-629 Warsaw, Poland; mmajder@sgsp.edu.pl

**Keywords:** vitamin D treatment, idiopathic hypercalciuria, urolithiasis, children, bone density

## Abstract

Vitamin D supplementation in patients with urolithiasis and hypercalciuria is considered to be unsafe. We analyzed the impact of vitamin D supplementation on selected health status parameters in children with idiopathic hypercalciuria. The study included 36 children with urolithiasis resulting from excessive calcium excretion. The level of calcium and 25(OH)D (hydroxylated vitamin D - calcidiol) in serum, urinary calcium excretion and the presence of stones in urinary tract were assessed prospectively. Blood and urine samples were collected at the time when the patient was qualified for the study and every three months up to 24 month of vitamin D intake at a dose of 400 or 800 IU/day. At time zero and at 12, and 24 months of vitamin D supplementation, densitometry was performed. Supplementation with vitamin D caused a statistically significant increase in the concentration of 25(OH)D in serum. There were no significant changes in calcium concentration in serum, excretion of calcium in urine but also in bone density. There was no significant increase in the risk of formation or development of stones in the urinary tract. Supplementation with vitamin D (400–800 IU/day) in children with idiopathic hypercalciuria significantly increases 25(OH)D concentration, does not affect calciuria, but also does not improve bone density.

## 1. Introduction

Vitamin D is a fat-soluble steroid hormone, which regulates calcium and phosphate metabolism. The skin exposed to UVB radiation produces pre-vitamin D, which binds to the DBP protein (vitamin D binding protein), and is transported to the liver cells, where is hydroxylated to 25(OH)D (calcidiol). Subsequently, in the proximal tubules of the kidney, the 1α-hydroxylase 25(OH)D converts it to 1.25(OH)D (calcitriol). The production of 25(OH)D unlike 1.25(OH)D is not strictly regulated. Due to the relatively long half-life (about 3 weeks) and chemical stability, this metabolite is an indicator of the level of vitamin D resources in the organism [1,2]. Patients with idiopathic hypercalciuria and urolithiasis were until recently included in the group of people whose supplementation with vitamin D is considered unsafe due to the possibility of increased calciuria and the formation of new stones in the urinary tract [3].

Idiopathic hypercalciuria (IH) is one of the most common metabolic causes of urolithiasis, both in children and adults (30–60% adults and 40–80% children). Urinary calcium excretion in children is considered increased when above 4 mg/kg body weight/24 h. [4]. The calcium-creatinine (Ca/Cr) ratio, calculated from the second morning urine sample, can also be used to estimate the level of hypercalciuria. The reference values of this indicator depend on age and range from 0.8 in infants to 0.2 in adults [4]. Symptoms of hypercalciuria are non-characteristic, including abdominal pain, haematuria, erythrocyturia [5,6,7]. The hypercalciuria may be the cause of formation of stones in urinary tract.

Urolithiasis (UL) is a condition in which in the urinary tract deposits are formed from chemicals that are normal or pathological constituents of urine [8,9,10,11]. In Europe, the incidence of this disease is estimated at about 4% in the adult population and 1–2% in children [8,12].

Both urolithiasis and hypercalciuria predispose to skeletal mineralization disorders, leading to a decreased bone density [8,9]. The human skeletal system is constantly changing. The most significant period is childhood and adolescence. Then, there is a rapid increase in the structure of the bone skeleton, which continues until the bone epiphysis is closed, i.e., until the age of 20. By this time, approximately 90% of bone mass is formed. The peak of bone mass is reached around 30 years of age. If insufficient, it is associated with an increased risk of osteoporosis in later life [9,13].

The gold standard in diagnosing bone density disorders is densitometry. In children, the bone density of the whole body is measured. The test result is expressed by means of indicators comparing the bone density of the tested person with the bone density of healthy people (Z-score). Z-score between (−2) and (−1) is considered as osteopenia in children, while osteoporosis is diagnosed at Z score > (−2), and accompanying clinical symptoms [1,9]. 

The identification of patients who are at risk of osteopenia and osteoporosis among patients with urolithiasis and idiopathic hypercalciuria allows the implementation of a preventive strategy that includes appropriate supplementation with vitamin D [1,14].

In this study, we evaluated the effect of vitamin D supplementation in children with idiopathic hypercalciuria on 25(OH)D blood level, caciuria, development of new stones in urinary tract and bone mineral density. 

## 2. Materials and Methods

### 2.1. Patients

The research project was approved by Bioethics Committee at the Military Institute of Medicine (Resolution No. 26/WIM/2013 of 22 May 2013). The study included 36 children (18 boys, 18 girls) with urolithiasis in the course of idiopathic hypercalciuria and low levels of vitamin D, hospitalized in Pediatrics, Nephrology, and Paediatric Allergology Department of Military Institute of Medicine. Inclusion criteria were age 5–16, urolithiasis in the course of idiopathic hypercalciuria, good cooperation with medical staff. Exclusion criteria were chronic kidney disease, urinary tract infections, urinary tract defects, systemic diseases, bone diseases (except osteopenia), endocrine disorders, and patients treated with glucocorticosteroids.

The written consent of legal guardians was obtained and, in the case of children over 15 years of age, also the consent of the child.

### 2.2. Experimental Study

**Figure 1:**
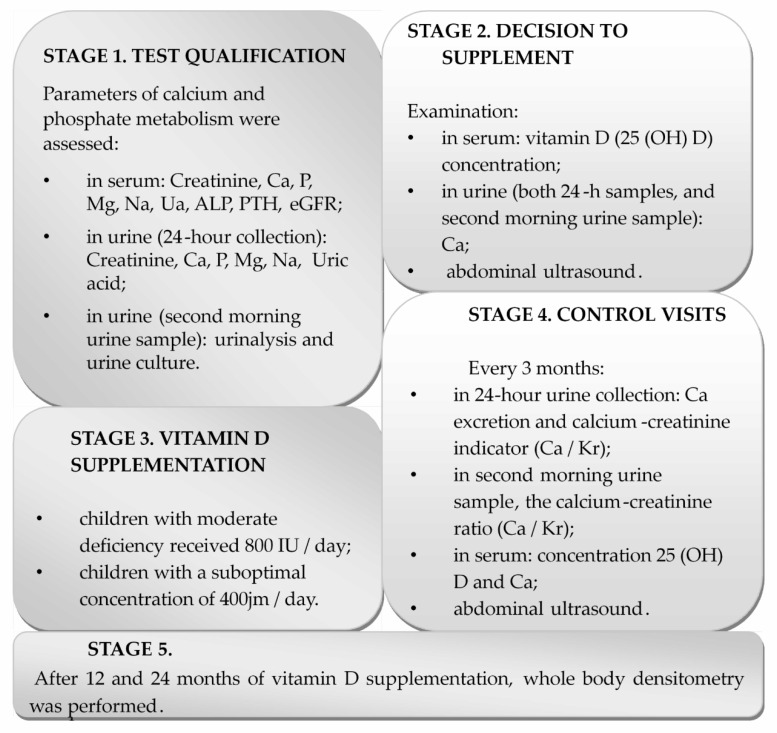
Study stages.

The medical interview included information about the onset of the problem with urolithiasis, accompanying diseases, and medications. All studied children were subjected to physical examination. The study was performed in four stages (Figure 1). Seven patients were followed up for 1 year, and 29 children for 2 years. The 1 year observation period was associated with reaching adulthood during the study period or resigning from the study after a year of observation.

Studied patients were supplemented with cholecalciferol tablets. We recommended to take the pill with a meal. During the study, vitamin D doses were adjusted to the season of the year and serum 25(OH)D concentrations (400 IU or 800 IU vitamin D dose) (Figure 1). Urolithiasis activity was assessed by ultrasound examination of the urinary system (no stones vs. new ones). In all cases in which calciuria would increase significantly during the treatment, intention was returning the patient to previous treatment and discontinuation of vitamin D.

### 2.3. Biochemical Parameters

Parameters, others than calcium, creatinine and 25(OH)D, examined during Stage 1 has been used to confirm diagnosis of idiopathic hypercalciuria.

Blood was collected from cubital vein to biochemical tubes (BD Falcon, Warsaw, Poland), and serum was obtained by the centrifugation method (20 min., 4 °C, 2000× *g*).

24-h and second morning urine samples were collected to the sterile container, and urinalysis and urine culture were performed. The urine samples were centrifuged (20 min., 4 °C, 2000× *g*) and the supernatant was collected for biochemical analysis. The concentration of calcium, phosphorus, magnesium, and sodium was measured by the photometric absorption method in Cobas 6000 analyzer (Roche Diagnostics, Warsaw, Poland). Creatinine and urea concentration in serum and urine were evaluated by the enzymatic method in Cobas 6000 analyzer. eGFR (estimated glomerular filtration rate was calculated using the Schwartz formula (0.413 × height (cm)/serum creatinine (mg/dL)). A result above 90 mL/min/1.73 m^2^ was considered as normal.

The Dia-Sorin LIAISON^®^ analyzer (Dia-Sorin, Saluggia, Italy) and chemiluminescent immunoassays (CLIA) were used to determine the concentration of total 25-hydroxy-vitamin D in serum and plasma. The values below 30 ng/mL indicated 25(OH)D deficiency.

Calciuria was assessed on the basis of calcium excretion in a 24-h urine sample (urine calcium concentration (mg/dL) × body weight (kg)/collection volume (dL)) and calcium/creatinine ratio (Ca/Cr) calculated from the second morning urine sample (urine calcium (mg/dL)/ urine creatinine (mg/dL). Calciuria was defined as calcium excretion above 4 mg/kg/day, the values of calcium-creatinine index indicating hypercalciuria depending on age (Table 1).

### 2.4. Ultrasonography and Densitometry

Abdominal ultrasound examinations were performed with the use of GE Logiq 5 Expert (Warsaw, Poland) and Philips EPIQ 5G (Warsaw, Poland) equipment. In all children, the abdominal ultrasound examination was performed according to the same examination protocol assessing the length and echogenicity of the kidneys, the presence/absence of dilatation of the calico-pelvic systems, and the presence/absence of urinary tract stones.

The densitometry was performed with the HOLOGIC model Delphi W (S/N 70608) in the Whole Body projection.

### 2.5. Statistical Analysis

Statistical evaluation of the results was performed using T-tests and one-way ANOVA with Bonferroni correction (in the case of a normal distribution) or non-parametric Kruskal–Wallis and Mann–Whitney U tests (in the case of an abnormal distribution). The data distribution was evaluated using the Shapiro–Wilk test. The percentile data were analyzed by the χ^2^ tests with modifications or the Fischer test, depending on the size of the subgroups. Correlation analysis and the related regression analysis were performed for variables whose relationships could have medical significance. The Statistica software (version 13.1; StatSoft, Cracov, Poland)) was used. The *p* < 0.05 was considered as statistically significant.

## 3. Results

### 3.1. Study Population

36 children (18 boys, 18 girls) aged 5–16 (mean 10.47 ± 3.41) were examined. Seven patients were followed for one year, the rest (29 children) for 2 years. Vitamin D deficiency (concentration < 30 ng/mL) was observed in all children (Table 2). At the beginning of the study, 13 children had stones in the urinary tract visible on ultrasound.

### 3.2. Vitamin D Level

**Figure 2:**
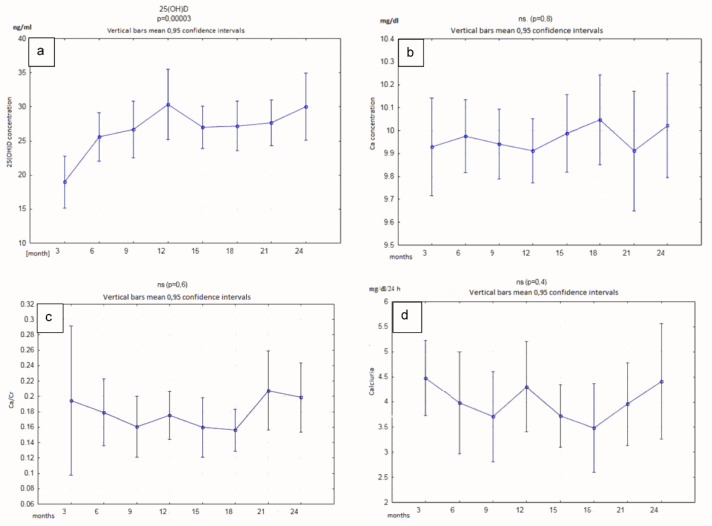
Time-dependent serum concentrations of vitamin D (**a**), calcium (**b**), and Ca/Creatinine (**c**), determined in the second morning urine sample, converted to creatinine ratio, and calciuria (**d**) determined in 24 h urine collection, in patients receiving vitamin D supplementation.

**Figure 3:**
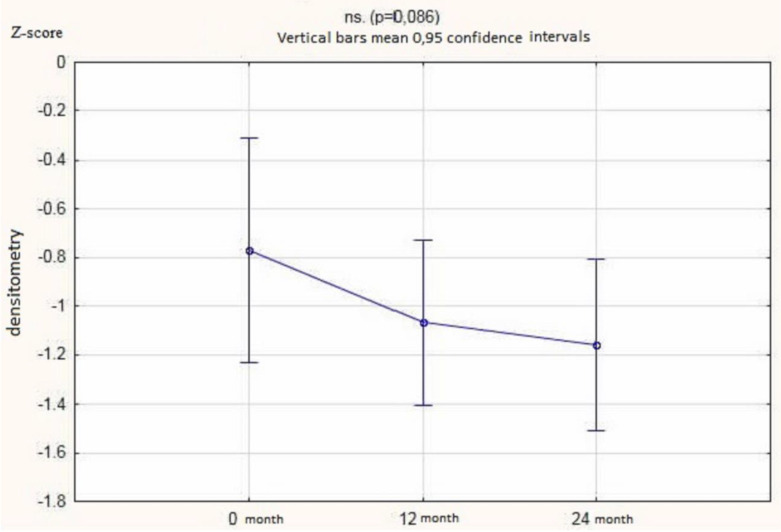
Z-score values in patients who received 12 or 24 months of vitamin D supplementation.

The vitamin D 25 (OH) D concentration in the serum determined after 3, 6, 9, 12, 15, 18, 21, and 24 months showed a statistically significant increase. We did not observe significant changes in the serum calcium concentrations or urinary calcium excretion (measured in mg/kg/24 h and with the use of calcium-creatinine ratio) (Figure 2 and Table 3). There were no significant changes in bones density measured by the Z-score after 12 or 24 months of supplementation (Figure 3).

### 3.3. Correlations

**Figure 4:**
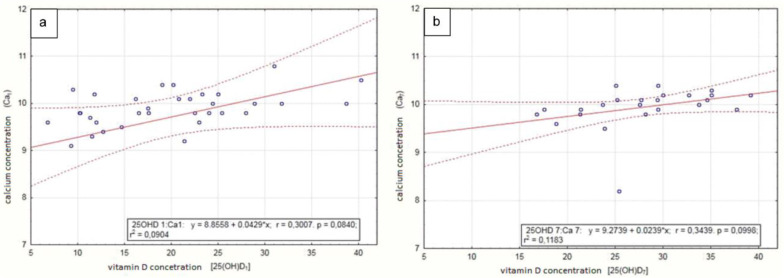
Dependence of serum calcium concentration on vitamin D concentration at 3 (**a**) and 21 (**b**) months after starting vitamin D administration.

A statistically significant correlation was found between vitamin D concentration and calcium concentration in the serum after 3 and 21 months of vitamin D supplementation. The average calcium concentration increased from 9.72 ± 1.19 to 10.06 ± 0.38 mg/dLwhich was still in the normal range), and the vitamin D concentration increased from 20.02 ± 8.52 to 27.89 ± 6.24 ng/mL. There was no statistically significant correlation between vitamin D and calcium concentration in the serum at other time points (Table 4, Figure 4). There was also no statistically significant correlation between vitamin D concentration and calcium excretion (in daily urine collection and in second morning urine sample, assessed by creatinine level) as well as between vitamin D concentration and Z-score assessed by densitometry (Table 4). The formation of new stones in the urinary system or enlargement of existing stones was not observed in the ultrasound examination performed during each follow-up visit (every 3 months) in children supplemented with vitamin D (Table 5).

## 4. Discussion

Interest in vitamin D and its properties has increased significantly over the past two decades. Numerous studies conducted in various regions of the world indicate that vitamin D deficiencies occur in all countries, regardless of the latitude and age [15,16,17,18]. This is also true for Polish children. In a multicentre study of the pediatric population, a clear deficiency of vitamin D was found in 75–80% of children [19].

Similar observations were made at the Department of Paediatrics, Nephrology, and Paediatric Allergology of Military Institute of Medicine, especially in children with idiopathic hypercalciuria and urolithiasis. These observations were the basis of the presented study. Vitamin D was administered to children with urolithiasis in the course of idiopathic hypercalciuria. This is one of the few studies on the use of vitamin D treatment in such group of patients. There is controversy on the effect of vitamin D administration on the urolithiasis activity. Earlier reports suggested the possibility of negative impact of vitamin D supplementation on the activity of urolithiasis, and vitamin D treatment in these patients was, therefore, contraindicated [20,21].

Despite physiological bases some authors have already demonstrated that vitamin D supplementation does not increase calciuria. Increased calcium reabsorption in the renal tubules, associated with increased serum vitamin D levels, should reduce urinary calcium excretion [1,4]. Therefore, we decided to use vitamin D in the doses lower than those normally used in the deficient population (ie. 400 IU/day or 800 IU/day). In case of a significant calciuria increase and/or increase in urolithiasis activity, the vitamin D administration was planned to be stopped. However, this problem did not occur in any of our study patients. The dose of vitamin D was determined based on serum 25(OH)D concentration. The dose of 400 IU/day of vitamin D was administered to patients with serum 25(OH)D concentrations > 20 ng/mL and a dose of 800 IU/day in patients with serum 25(OH)D < 20 ng/mL concentration. There are no clear recommendations in the world literature regarding the administration of vitamin D in patients with low bone density and idiopathic hypercalciuria [4,9,20]. All known studies were performed in adult population and results are often contradictory. The observations from our study confirmed the observations of Ticinesi et al. [22] showing the decreased serum vitamin D concentration in patients with urolithiasis. The authors reported the patients with urolithiasis had a lower concentration of vitamin D than the patients from the control group (without urinary problems). They even concluded that vitamin D deficiency (<20 ng/mL) increases the risk of calcium stones.

Many studies have been conducted to assess the safety of vitamin D supplements in healthy people. Most studies have shown no adverse effects, either with constant use or with single high dose of vitamin D [23,24,25,26,27,28]. However, most of the analyzed cases were postmenopausal women or people with accompanying chronic diseases, including lupus, renal failure, diabetes, multiple sclerosis, or respiratory diseases, the course of which may affect the results. None of such analyzes were performed in children. In the present study, we showed, that vitamin D administration correlated with a statistically significant increase in the serum concentration of the hepatic metabolite of vitamin D. This result was consistent with other studies [29,30,31,32,33,34]. We also showed that low doses of vitamin D (400 IU/day) in patients with D hypovitaminosis caused a significant increase in the concentration of 25(OH)D in the serum. We also showed that there was no significant effect of vitamin D administration on calciuria and there was no correlation between concentration of vitamin D and calciuria, which is in agreement with the study of Eisner et al. [31]. Similar results were also observed by other authors. Penntiston et al. [34] showed that supplementation with high doses of ergocalciferol (vitamin D2) in healthy postmenopausal women, including four postmenopausal women with hypercalciuria, did not increase calcium excretion in urine. Similar results were obtained by Leaf et al. (29) who showed that in adult population with urolithiasis there was no significant increase in calcium excretion, despite the use of high doses of ergocalciferol (50,000 IU per week) for 2 months. Also, Johri et al. (30) found no statistically significant increase in calcium excretion in urine after 2 months supplementation with vitamin D (20,000 IU per week) in adults with urolithiasis, and vitamin D deficiency (<12 ng/mL in serum). However, in the group of patients with a low level of calciuria, there was an increase of calcium excretion in urine, which could have been associated with latent idiopathic hypercalciuria, and mutation or polymorphism in CYP24A1 gene. Also, Letaverier et al. [33] found a higher level of calcium extraction in the urine of rats supplemented with vitamin D. The calciuria was enhanced by the administration of calcium [33].

In the present study, the children’s diet contained a normal concentration of calcium, and children did not receive additional calcium supplementation. Their calcium serum concentration was within the normal range. We found a statistically significant correlation between the concentration of calcium and vitamin D in the serum after 3 and 21 months of vitamin D administration. We also found that there was no increase in the activity of urolithiasis (defined as the presence of new stones in the urinary tract), assessed by the ultrasound analysis performed every 3 months. Similar results were obtained by Ferraro et al. [35] who evaluated the influence of vitamin D treatment on the increased risk of urolithiasis in almost 200,000 medical professionals. They divided the study group based on the daily intake (from 100 to 1000 IU) of vitamin D [35]. They found that vitamin D supplementation did not increase the risk of kidney stones. However, a limitation of the study was the method of assessing the activity of urolithiasis, which was solely based on reported renal colic incidents. In contrast, Letavernier et al. [33] showed the higher activity of stone formation in the group receiving calcium with vitamin D compared to the group receiving calcium alone, vitamin D alone, or the control group.

Bone mass is built in the first three decades of life and after the reached peak, involution of bone mass begins. The size of bone peak mass is fundamental for the risk of developing osteoporosis in the future [36,37]. The correlation between hypercalciuria and urolithiasis and osteopenia and osteoporosis is well documented [31,38]. In children, it is probably associated with increased calcium resorption from bone and/or increased bone turnover [1]. However, there is still no clear opinion on the effect of vitamin D supplementation on bone density in children with urolithiasis and hypercalciuria. The studies of Garcia-Nieto et al. [39] on the population of children with idiopathic hypercalciuria found osteopenia in 30% of the respondents (22 out of 73 children). In children with reduced bone density, they observed lower excretion of citrates and higher excretion of uric acid, which could affect both the activity of urolithiasis and the bone calcification state [39]. A comparison of 88 children with idiopathic hypercalciuria with 29 healthy children by Penido et al. [40] showed reduced bone mineral density in the lumbar spine in 35% of children with idiopathic hypercalciuria. Schwaderer et al. [41] observed a higher risk of bone mineralization disorders in children with idiopathic hypercalciuria and active urolithiasis than in children with idiopathic hypercalciuria alone. They also noted that such disorders more often affected boys with an increased Body Mass Index (BMI). On the other hand, Artemiuk et al. [42] showed a correlation between low vitamin D concentrations and reduced bone density in the lumbar spine of children. They also drew attention to the more frequent occurrence of bone mass reduction in older children compared to the younger group (mean age 11 vs. 8.5 years). In the present study, there was no improvement in bone density parameters in a densitometric examination, measured with Z-score, after 12 and 24 months supplementation with vitamin D.

Adequate dietary calcium intake is important for both urolithiasis course and bone density. Both too little and too much calcium supply in the diet favors the formation of stones in the urinary tract. According to the current opinion, patients diagnosed with idiopathic hypercalciuria should be switched to the normocalcemic diet [43]. On the other hand, patients with low bone mass are recommended a high-calcium diet, which, as previously shown, may promote the greater activity of urolithiasis. In the present work, we showed that the supplementation with low doses of vitamin D for 24 months together with the normocalcium diet is insufficient to improve bone mass. The lack of improvement in bone mineral density parameters is probably related to too low doses of vitamin D or too short observation period. One of the most important conclusions from our work is that the administration of the 400 or 800 IU/day of vitamin D seems to be safe as it did not affect the severity of urolithiasis activity.

## 5. Limitation

We are aware, however, that our study had some limitation: 1. the size of the studied group of children was small, 2. we had not full control of the diet or 3. regular administration of vitamin D. In our opinion the study of a larger population of children with supplementation with higher doses of vitamin D should be performed. In other limitations, we assumed that a three-month biochemical evaluations of patients were sufficient to conclude that patients followed with the recommendations.

## 6. Conclusions

(1). Supplementation with low doses of vitamin D (400–800 IU/day) in children with idiopathic hypercalciuria significantly increases the concentration of 25OHD vitamin D in the serum and does not affect the level of calciuria. It does not increase the dynamics of stones formation in the urinary tract, but also does not improve bone density.

(2). The use of vitamin D preparations in these patients is safe, without a significant influence on the severity of disease activity.

(3). Children with idiopathic hypercalciuria should be advised to carefully monitor the parameters of calcium metabolism and the level of urolithiasis activity without giving up vitamin D supplementation.

## Figures and Tables

**Table 1:** Values of the calcium-creatinine index in children, indicative of hypercalciuria.

Age	mg Calcium/mg Creatinine
<1 year	<0.81
1–3 years	<0.53
3–5 years	<0.39
5–7 years	<0.28
>7 years	<0.21

**Table 2:** Mean values of the assessed parameters at the beginning of the study.

Parameter	N	Mean ± SD	Units	Median (q25–q75)
25-hydroxy-vitamin D	35	20.02 ± 8.52	ng/mL	20.2 (11.8–25.00)
Ca	35	9.72 ± 1.19	mg/dL	9.8 (9.6–10.20)
Calciuria	36	4.28 ± 1.85	mg/kg/24 h	4.515 (2.94–5.37)
Ca/Creatinine	33	0.19 ± 0.16	mg/mg	0.198 (0.13–0.26)
Z-score	36	−0.73 ± 0.87	-	−0.855 ((−)1.26–(−)0.36)

**Table 3:** The mean values of the assessed parameters after 24 months of vitamin D supplementation.

Parameter	N	Mean ± SD	Units	Median (q25–q75)
25-hydroxy-vitamin D	22	29.85 ± 9.65	ng/mL	29.25 (21.5–35.5)
Ca	21	10.02 ± 0.41	mg/dL	9.9 (9.8–10.20)
Calciuria	21	4.5 ± 2.24	mg/kg/24h	4.06 (3.30–5.43)
Ca/Creatinine	22	0.2 ± 0.09	mg/mg	0.185 (0.13–0.26)
Z-score	23	−1.05 ± 0.77	-	−0.96 ((−)1.7–(−)0.56)

**Table 4:** Correlations between vitamin D and calcium concentration in the serum, and calcium excretion (in the 24-h and the second morning urine sample, assessed by creatinine level) at different time points. The statistically significant values are marked in bold.

Variables	Time	R Spearman	*p*
25-hydroxy-vitamin D and calcium (Ca)	3 months	0.451871	0.007301
	6 months	0.215881	0.220112
	9 months	0.216470	0.226283
	12 months	0.037120	0.834899
	15 months	0.297773	0.103748
	18 months	0.068840	0.743692
	21 months	0.560887	0.004355
	24 months	0.237260	0.300404
25-hydroxy-vitamin D and calciuria	3 months	0.093781	0.592064
	6 months	−0.040049	0.822074
	9 months	−0.016993	0.921642
	12 months	0.223403	0.204067
	15 months	0.059459	0.754959
	18 months	0.087581	0.670520
	21 months	−0.142049	0.507896
	24 months	−0.059740	0.797001
25-hydroxy-vitamin D and Ca/creatinine ratio	3 months	−0.039043	0.831986
	6 months	−0.040196	0.821432
	9 months	−0.006245	0.971165
	12 months	−0.133705	0.450932
	15 months	0.176983	0.332539
	18 months	−0.098432	0.647248
	21 months	−0.235857	0.267205
	24 months	−0.206331	0.356917
25-hydroxy-vitamin D and densitometry	12 months	0.104499	0.575855
	24 months	−0.314512	0.153997

**Table 5:** The relationship between vitamin D concentration and urolithiasis activity after 3, 12, and 24 months of vitamin D supplementation.

	Time	*p* Value	Odds Ratio	Confidence OR −95%	Confidence OR +95%
25-hydroxy-vitamin D and urolithiasis activity	3 months	0.761	0.987	0.909	1.072
	12 months	0.498	1.027	0.952	1.108
	24 months	0.509	1.039	0.927	1.164
